# Real-Time EEG-Based Happiness Detection System

**DOI:** 10.1155/2013/618649

**Published:** 2013-08-18

**Authors:** Noppadon Jatupaiboon, Setha Pan-ngum, Pasin Israsena

**Affiliations:** ^1^Department of Computer Engineering, Faculty of Engineering, Chulalongkorn University, Bangkok 10330, Thailand; ^2^National Electronics and Computer Technology Center, Pathumthani 12120, Thailand

## Abstract

We propose to use real-time EEG signal to classify happy and unhappy emotions elicited by pictures and classical music. We use PSD as a feature and SVM as a classifier. The average accuracies of subject-dependent model and subject-independent model are approximately 75.62% and 65.12%, respectively. Considering each pair of channels, temporal pair of channels (T7 and T8) gives a better result than the other area. Considering different frequency bands, high-frequency bands (Beta and Gamma) give a better result than low-frequency bands. Considering different time durations for emotion elicitation, that result from 30 seconds does not have significant difference compared with the result from 60 seconds. From all of these results, we implement real-time EEG-based happiness detection system using only one pair of channels. Furthermore, we develop games based on the happiness detection system to help user recognize and control the happiness.

## 1. Introduction

The aim of human computer interaction (HCI) is to improve the interactions between human and computers. Because most computers lack of understanding of user's emotions, sometimes they are unable to respond to the user's needs automatically and correctly [1]. One of the most interesting emotions is happiness. world happiness report reflects a new worldwide demand for more attention to happiness and absence of misery as criteria for government policy [2]. Being happy is related to many positive effects including confidence, optimism, self-efficacy, likability, activity, energy, physical well-being, flexibility, creativity, and the ability to cope with stress [3]. All of these benefits are the reasons why we should be happy.

In the past decades, most of emotion recognition researches have only focused on using facial expressions and speech. However, it is easy to fake facial expressions or change tone of speech and these signals are not continuously available, and they differ from using physiological signals, which occur continuously and are hard to conceal, such as Galvanic Skin Response (GSR), Electrocardiogram (ECG), Skin Temperature (ST), and, especially, Electroencephalogram (EEG). EEG is the signal from voltage fluctuations in the brain, that is, the center of emotions [1, 4]. Emotions are thought to be related with activity in brain areas that direct our attention, motivate our behavior, and determine the significance of what is going on around us. Emotion is related with a group of structures in the center of the brain called limbic system, which includes amygdala, thalamus, hypothalamus, and hippocampus [5, 6]. 

**Figure 1:**
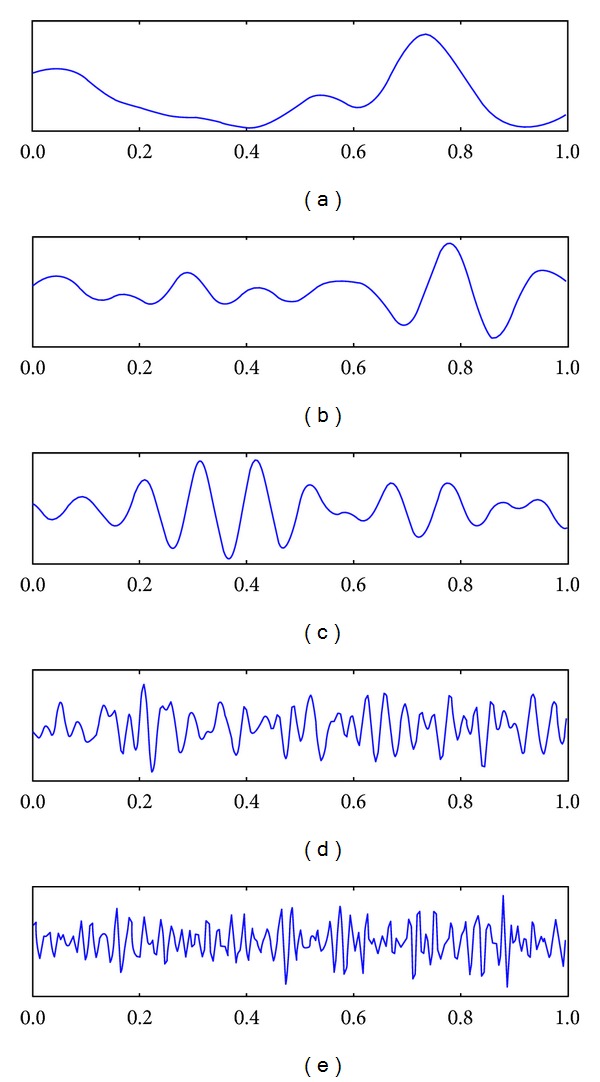
Brainwave: (a) Delta, (b) Theta, (c) Alpha, (d) Beta, and (e) Gamma [9].

**Figure 2:**
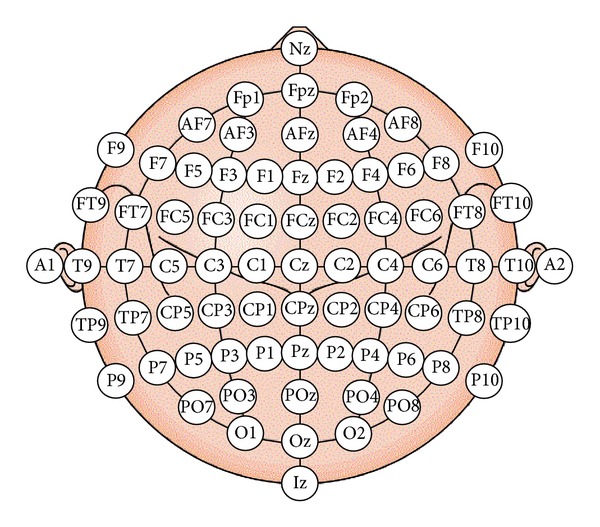
International 10–20 system of electrode placement [7].

Electroencephalogram (EEG) is the recording of electrical activity on the scalp. EEG measures voltage changes resulting from ionic current flows within the neurons of the brain. There are five major brain waves distinguished by their different frequency bands (number of waves per second) as shown in Figure 1. These frequency bands from low to high frequencies, respectively, are called Delta (1–3 Hz), Theta (4–7 Hz), Alpha (8–13 Hz), Beta (14–30 Hz), and Gamma (31–50 Hz).  Figure 2 shows the 10–20 system of electrode placement, that is, an internationally recognized method to describe and apply the location of scalp electrodes. Each site has a letter to identify the lobe and a number to identify the hemisphere location [7, 8].

## 2. The Literature Review

Nowadays, the EEG-based emotion recognition researches are highly active. The goal of these is to find suitable technique giving a good result that eventually can be implemented in real-time emotion recognition. The list of the EEG-based emotion recognition researches is shown in Table 1. It is difficult to compare results among them because there are a lot of factors that make different results from different researches including participant, model of emotion, stimulus, feature, temporal window, and classifier. The main six factors are described next to clarify the understanding. 

### 2.1. Participant

The larger number of participants makes more reliable result. Moreover, we can divide the method for building emotion classification into subject-dependent and subject-independent models. The second model is harder than the first model due to interparticipants variability [10, 11]. The subject-dependent model avoids the problems related to interparticipant but a new classification model must be built for every new user. In this research, we build both subject-dependent and subject-independent models to compare the results.

### 2.2. Model of Emotion

**Figure 3:**
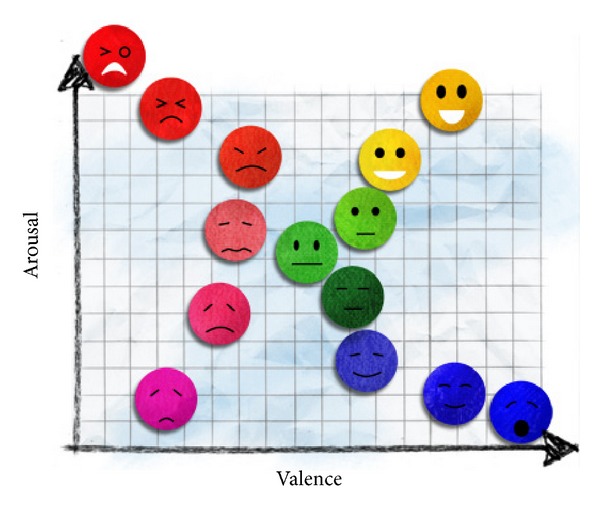
Dimensional model of emotion [14].

The larger number of emotions makes emotion recognition harder, and some emotions may overlap. A good model of emotion should clearly separate these emotions. Several models have been proposed such as basic emotion and dimensional model. The most widely used basic emotions are the 6 basic emotions (i.e., anger, disgust, fear, joy, sadness, and surprise) that have been mostly used in facial expression recognition [12]. The common dimensional model is characterized by two main dimensions (i.e., valence and arousal). The valence emotion ranges from negative to positive, whereas the arousal emotion ranges from calm to excited [13]. This model is used in most researches because it is easier to express an emotion in terms of valence and arousal rather than basic emotions that can be confused by emotion names [14]. As shown in Figure 3, the emotions in any coordinates of the dimensional model are shown by facial expression. In this research, we use the dimensional models. The emotions used are happy and unhappy (sad). The happy emotion has positive valence and low arousal whereas the unhappy emotion has negative valence and low arousal.

### 2.3. Stimulus

There are various methods for emotion elicitation, which are self-eliciting, recalling, and using external stimulus such as picture, sound, and odor. The widely used databases for emotion elicitation are International Affective Picture System (IAPS) [15] and International Digitized Sound System (IADS) [16]. These databases are generally accompanied by emotional evaluations from average judgments of several people. In this research, we choose pictures from Geneva Affective Picture Database (GAPED) [17] and sounds from classical emotion elicitation, because using visual-audio stimulus gives a better result than using either visual stimulus or audio stimulus [18].

### 2.4. Feature

Several signal characteristics of EEG have been used to be the features. The widely used feature is Power Spectral Density (PSD), the power of the EEG signal in focused frequency bands. In addition, others such as Spectral Power Asymmetry (ASM), Common Spatial Pattern (CSP), Higher Order Crossings (HOC), Self-Organizing Map (SOM), Higher Order Spectra (HOS), Fractal Dimension (FD), Asymmetric Spatial Pattern (ASP), and Entropy have been used as features and some give a good result. In this research, the feature we use is PSD since it gives a good performance in several researches as shown in Table 1, and it uses relatively little computation, which is suitable to implement in real-time emotion recognition. 

### 2.5. Temporal Window

The appropriate length of temporal window depends on a type of emotion and physiological signal. Overall duration of emotions approximately falls between 0.5 and 4 seconds [42]. By using unsuitable window, the emotion may be misclassified because different emotions may be covered when too long or too short periods are measured. The existing literature does not provide suitable window size to be used to achieve optimal EEG-based emotion recognition [4]. In this research, we use temporal window 1 second.

### 2.6. Classifier

Several machine learning algorithms have been used as emotion classifiers such as Support Vector Machine (SVM), Naïve Bayes (NB), Quadratic Discriminant Analysis (QDA), K-Nearest Neighbors (KNN), Linear Discriminant Analysis (LDA), and Multilayer Perceptron (MLP). As shown in Table 1, SVM is implemented on many emotion classification researches because of many advantages. SVM is known to have good generalization properties and to be insensitive to overtraining and curse of dimensionality. The basic training principle of SVM is finding the optimal hyperplane where the expected classification error of test samples is minimized. The optimal hyperplane is the one that maximizes the margins. Maximizing the margins is known to increase the generalization capability. SVM uses regularization parameter (C) that enables accommodation to outliers and allows errors on the training set [43]. In this research, we use Gaussian SVM to be a classifier. 

Beside the aforementioned factors, there is a factor that affects classification results from different researches. We found that some researches did not separate training set and test set completely although they did cross-validation (CV). Because simple cross-validation method randomly selects some data to be test set and the rest of data to be training set, some training data and test data may be in the same trial. Although the offline result is good, it does not guarantee the online result. In online emotion recognition, the training set is used to build the classification model, and the test set is a data from real-time EEG, so the training data and the test data are absolutely separated. For reliable result that can be guaranteed when using online emotion recognition, we should separate training set and test set completely. In this research, we use leave-one-trial-out cross-validation (LOTO-CV) and leave-one-subject-out cross-validation (LOSO-CV) for evaluating subject-dependent and subject-independent models, respectively.

As shown in Table 1, most of EEG-based emotion recognition researches are not for real-time implementation. There are a few researches that implement real-time emotion recognition such as [29, 40]. Wijeratne and Perera [40] proposed real-time emotion detection system using EEG and facial expression. However, the EEG signal acquisition part was still offline due to their time constraints, so they used pre-recorded EEG data instead of real-time EEG data. Liu et al. [29] proposed real-time emotion detection system using EEG. The user emotions are recognized and visualized in real time on his/her avatar. However, there is an issue in their approach that needs to be mentioned. In order to recognize an emotion, they did not use classifier and they only compared the Fractal Dimension (FD) values with predefined threshold, but they did not show how to define that threshold. 

To fulfill these, we intend to implement EEG-based emotion detection system that can be truly implemented in real-time. Due to real-time processing, minimum computation time is required. We compare results among each pair of channels and different frequency bands in order to reduce insignificant channels and frequency bands. Furthermore, we develop games based on the happiness detection system to recognize and control happiness. 

## 3. Methodology

**Figure 4:**
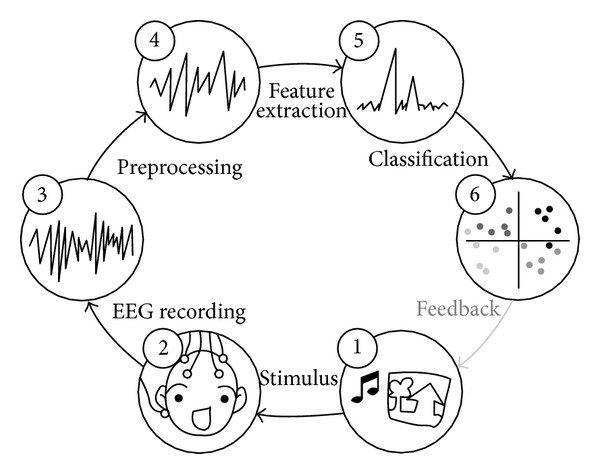
The process of emotion classification [19].

The process of emotion classification consists of several steps as shown in Figure 4. First of all a stimulus such as picture, audio, and movie is needed. During experiment, the participant is exposed to the stimuli to elicit emotion, and EEG signal is recorded accordingly. Then artifacts that contaminate EEG signal are removed. These EEG data are analyzed and relevant features are extracted. Some parts of data are trained to build classification model and the rest of data, which are test data, are classified using this model. 

### 3.1. Stimulus

Both pictures and classical music were used to be the stimulus to elicit emotion. For pictures from GAPED [17], we selected the 50 highest valence scored pictures to be happy stimulus (i.e., pictures of human and animal babies as well as nature sceneries) and the 50 lowest valence scored pictures to be unhappy stimulus (i.e., pictures of human concerns and animal mistreatments). For classical music, we selected the highest and lowest valence scored pieces according to Vempala and Russo [44] to be happy and unhappy stimuli, respectively. The happy and unhappy pieces were Tritsch Tratsch Polka by Johann Strauss and Asas' Death by Edvard Grieg, respectively.

### 3.2. EEG Recording

**Figure 5:**
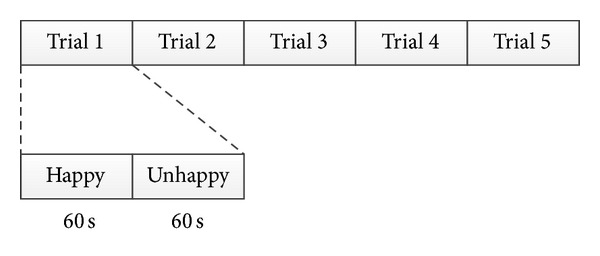
Procedure of experiment.

We used 14-channels wireless EMOTIV [45] (i.e., AF3, AF4, F3, F4, F7, F8, FC5, FC6, P7, P8, T7, T8, O1, and O2). The sampling rate is 128 Hz. The resolution is 16 bits (14 bits effective). Before recording EEG, we put EMOTIV on the participant's head for a while to prevent undesired emotions that can arise from unfamiliar or uncomfortable feelings. Then we described the process of recording and advised the participant to stay as still as possible to prevent artifact that can occur from moving the body. When the participant was ready, we then recorded EEG and the experiment was started. As shown in Figure 5, there were 5 trials, where each trial consisted of one happy and one unhappy stimulus. Each stimulus was composed of 10 pictures and 1 piece of classical music that played along for 60 seconds. After that, a blank screen was shown for 12 seconds to adjust participant's emotion to normal state and then the next stimulus was shown. When the 5 trials were completely shown, the process of recording ended. All these steps took approximately 15 minutes. There were 10 participants (i.e., 1 male and 9 females; average age is 34.60) taking part in this experiment.

### 3.3. Preprocessing

The EEG signal was filtered using a 5th-order sinc filter to notch out power line noise at 50 Hz and 60 Hz [45]. We removed baseline of the EEG signal for each channel so the values of the signal are distributed around 0.

### 3.4. Feature Extraction

The EEG signal with window 1 second was decomposed to 5 frequency bands that are Delta (0–4 Hz), Theta (4–8 Hz), Alpha (8–16 Hz), Beta (16–32 Hz), and Gamma (32–64 Hz) by Wavelet Transform as shown in Table 2. Then the PSD from each band was computed to be the feature. Since EMOTIV have 14 channels, the total features are 70. The features were normalized for each participant by scaling between 0 and 1 as shown in (1) to reduce interparticipant variability [11]:

(1) $$\begin{array}{c} \text{normalize }\left(X_{i}\right)=\frac{X_{i}-X_{\min}}{X_{\max}-X_{\min}}. \end{array}$$

Since EEG signal from each trial has 120 seconds, there are 120 samples per trial. Due to 5 trials, there are 600 samples per participant. With 10 participants, the total samples are 6000. All samples were labeled whether happy or unhappy depending on the type of stimulus.

### 3.5. Classification

Gaussian SVM with leave-one-trail-out cross-validation (LOTO-CV) and leave-one-subject-out cross-validation (LOSO-CV) were used to compute accuracy for subject-dependent and subject-independent models, respectively. In the LOTO-CV method with 5 trials, one trial is set to be a test set and the rest to be a training set. Then the training set is built to be a classification model and the test set is classified using this model to evaluate accuracy. After that, we repeated the process using different trials as test sets, until all of the 5 trials had been test sets. The accuracy reported is the average accuracy of all 5 trials. The appropriate parameters are the set giving the best average of the 5 accuracies. In the LOSO-CV method with 10 subjects, one subject is set to be a test set and the rest to be a training set. Then the training set is built to be a classification model and the test set is classified using this model to evaluate accuracy. After that, we repeated the process using different subjects as test sets, until all of the 10 subjects had been test sets. The appropriate parameters are the set giving the best average of the 10 accuracies. The appropriate parameters C and *γ* of SVM were selected by grid search method. SVM implementation was done using LIBSVM [46].

## 4. Results and Discussion

### 4.1. Subject-Dependent and Subject-Independent Models

**Figure 6:**
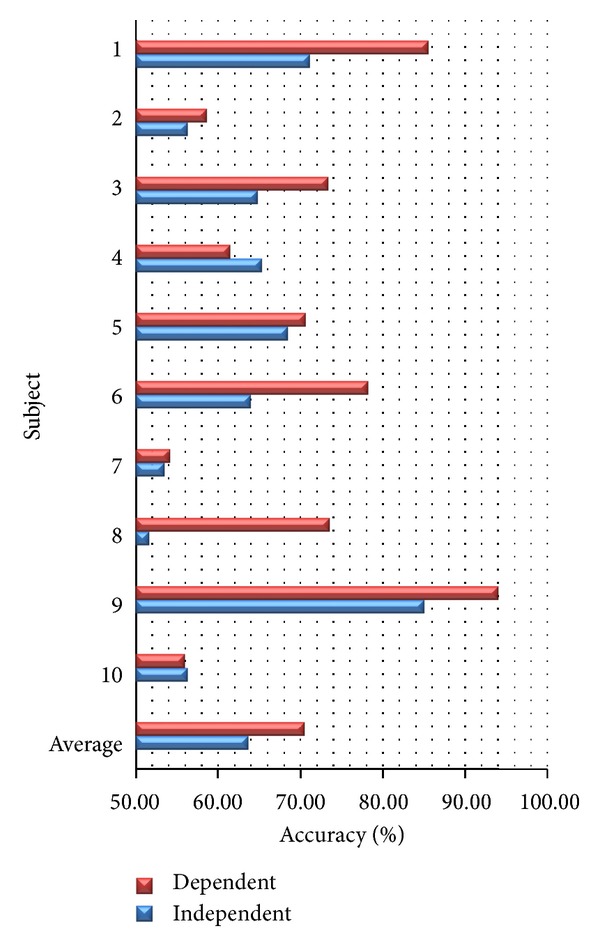
Accuracy from subject-dependent and subject-independent models.

We compare subject-dependent and subject-independent accuracies using all features. As shown in Figure 6, we found that most of subject-independent accuracies are lower than subject-dependent accuracies. The average accuracies of subject-dependent model and subject-independent model are 70.55% and 63.67%, respectively. We can conclude that there are a lot of interparticipants. Different subjects may have different patterns of EEG when emotions are elicited. This conclusion is consistent with [24, 36]. As a result, we use only subject-dependent model to implement on real-time happiness detection system. Furthermore, we found that all of the older subjects (i.e., subject 2, 4, and 10; average age is 57.50) are giving low accuracies (accuracy of subject-dependent model lower than 65%). All of them confirm that they were elicited well by stimulus. We suppose as Levenson et al. [47] found that the magnitude of change in physiological signal was smaller in older than in younger subjects during emotion elicitation. So the accuracies of older subjects are low. When we exclude these older subjects, the average accuracies of subject-dependent model and subject-independent model are up to 75.62% and 65.12%, respectively.

### 4.2. Varying Pairs of Channels

**Figure 7:**
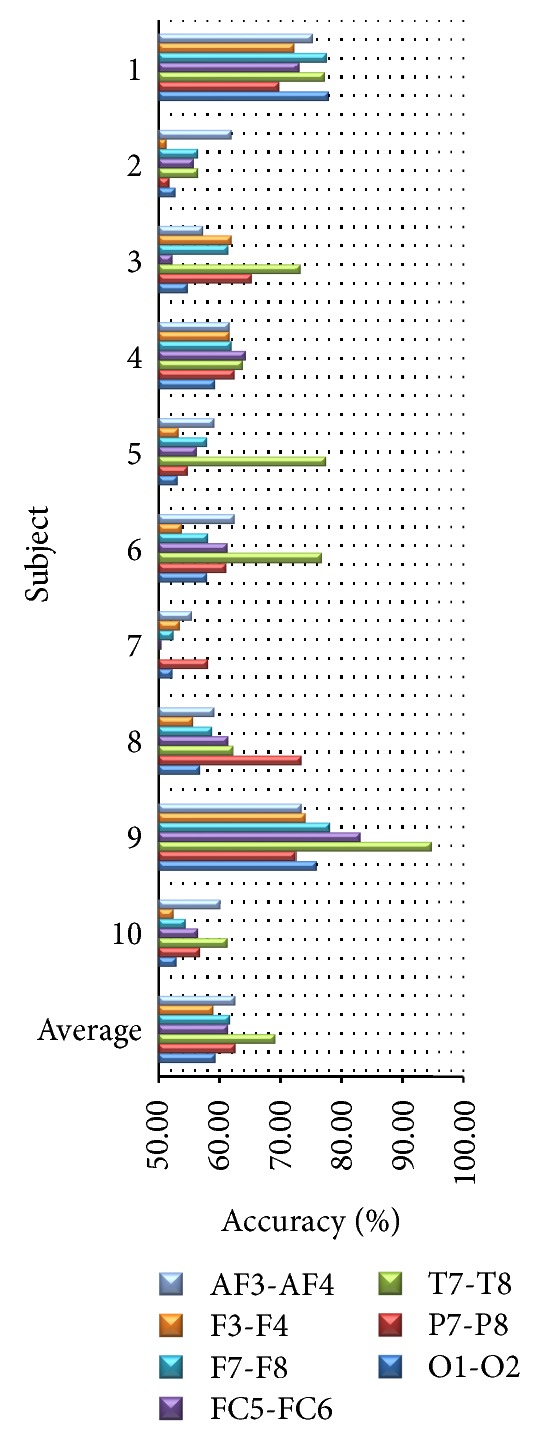
Accuracy from each pair of channels.

We compare subject-dependent accuracy among each pair of channels (i.e., AF3-AF4, F3-F4, F7-F8, FC5-FC6, P7-P8, T7-T8, and O1-O2) using all frequency bands. As shown in Figure 7, we found that the highest average accuracy at 69.20% given by the pair of T7-T8 is very close to the average accuracy given by all channels. When we exclude older subjects, the average accuracy of T7-T8 is still highest at 72.90%. With PSD feature, we can conclude that temporal lobe is more effective for classifying happy and unhappy emotions than the others. This conclusion is consistent with [35, 48]. As a result, we can use this pair of channels instead of fourteen channels to reduce the number of channels and save computation time. 

### 4.3. Varying Frequency Bands

**Figure 8:**
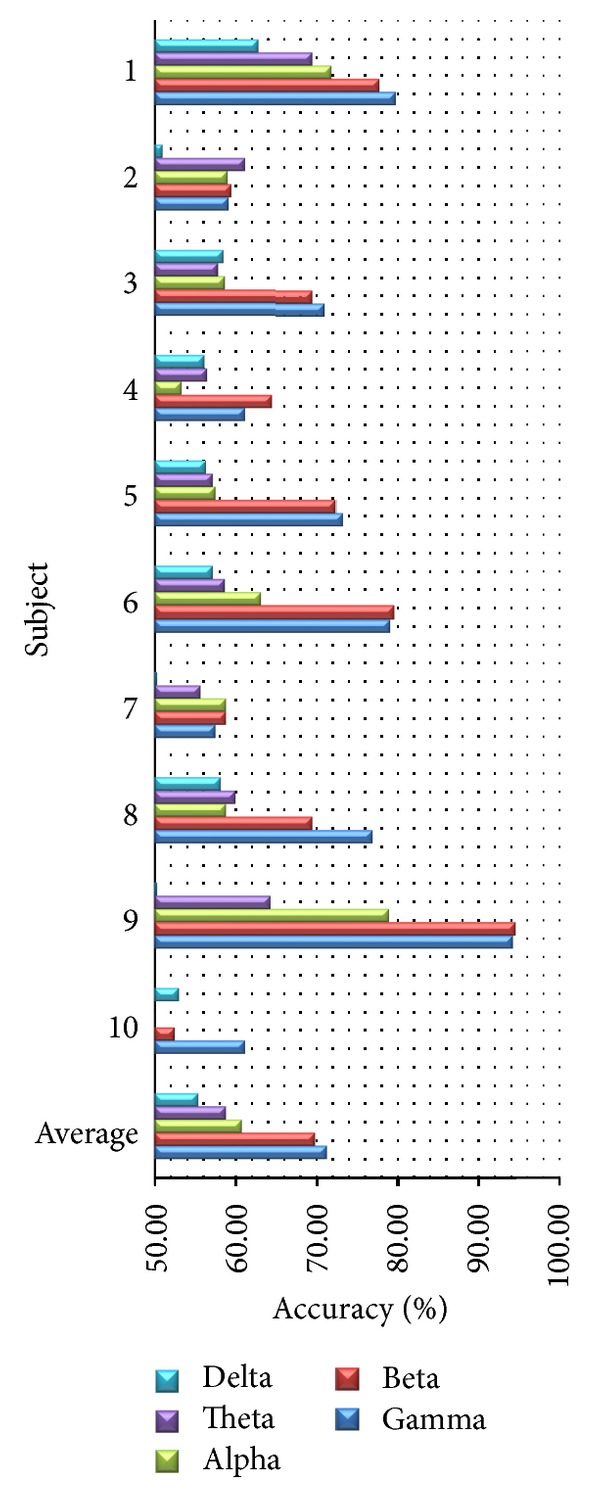
Accuracy from different frequency bands.

We compare subject-dependent accuracy among different frequency bands (i.e., Delta, Theta, Alpha, Beta, and Gamma) using all channels. As shown in Figure 8, we found that the average accuracies of Beta and Gamma are 69.83% and 71.28%, respectively, which are clearly higher than these of the other bands. When we exclude older subjects, the average accuracies of Beta and Gamma are still clearly higher than these of the other bands at 74.55% and 75.90%, respectively. With PSD feature, we can conclude that high frequency bands are more effective for classifying happy and unhappy emotions than low frequency bands. This conclusion is consistent with [20, 31, 48]. As a result, we can omit low-frequency bands such as Delta and Theta in order to save computation time. 

### 4.4. Varying Time Durations

**Figure 9:**
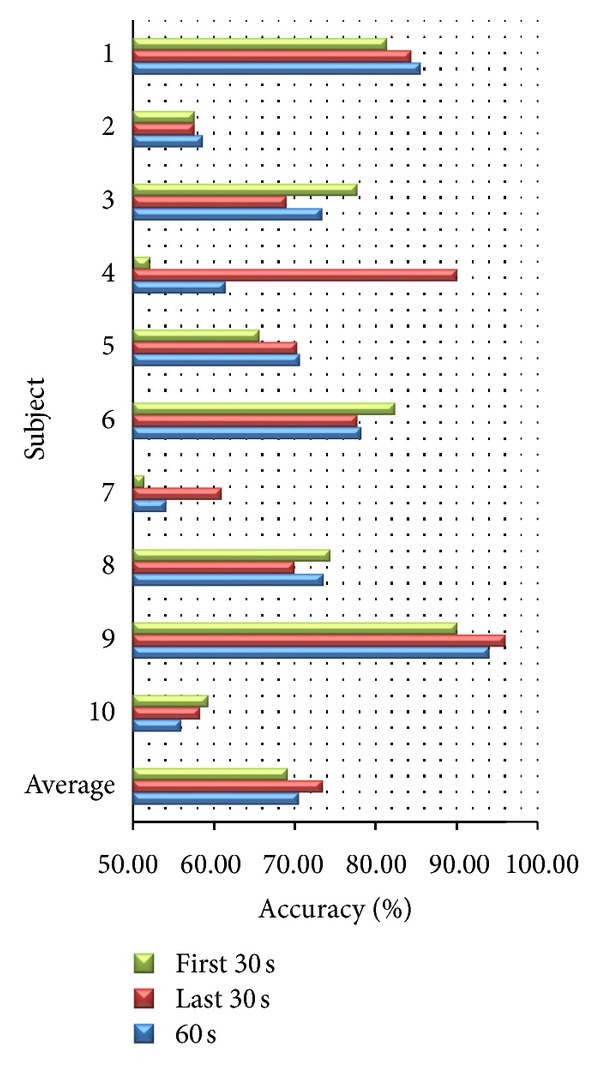
Accuracy from different time durations.

We compare subject-dependent accuracy from different time durations for emotion elicitation using all features. We consider accuracy from the first 30 seconds and the last 30 seconds of each stimulus. As shown in Figure 9, we found that the average accuracies of the first 30 seconds and the last 30 seconds are 69.17% and 73.43%, respectively. When we exclude older subjects, the average accuracies of the first 30 seconds and the last 30 seconds are up to 74.67% and 75.48%, respectively. Some subjects have higher accuracy in the first 30 seconds than the last 30 seconds and some subjects have higher accuracy in the last 30 seconds than the first 30 seconds. It shows that the time duration to elicit emotion is different depending on subjects. Considering statistical significance, we found that result from the first 30 seconds does not have significant difference from the result from the last 30 seconds (*P* value > 0.05). Furthermore, result from the first 30 seconds does not have significant difference from the result from 60 seconds (*P* value > 0.05). As a result, we may reduce time to elicit emotion from 60 to 30 seconds to save time duration for emotion elicitation.

## 5. Real-Time Happiness Detection System

**Figure 10:**
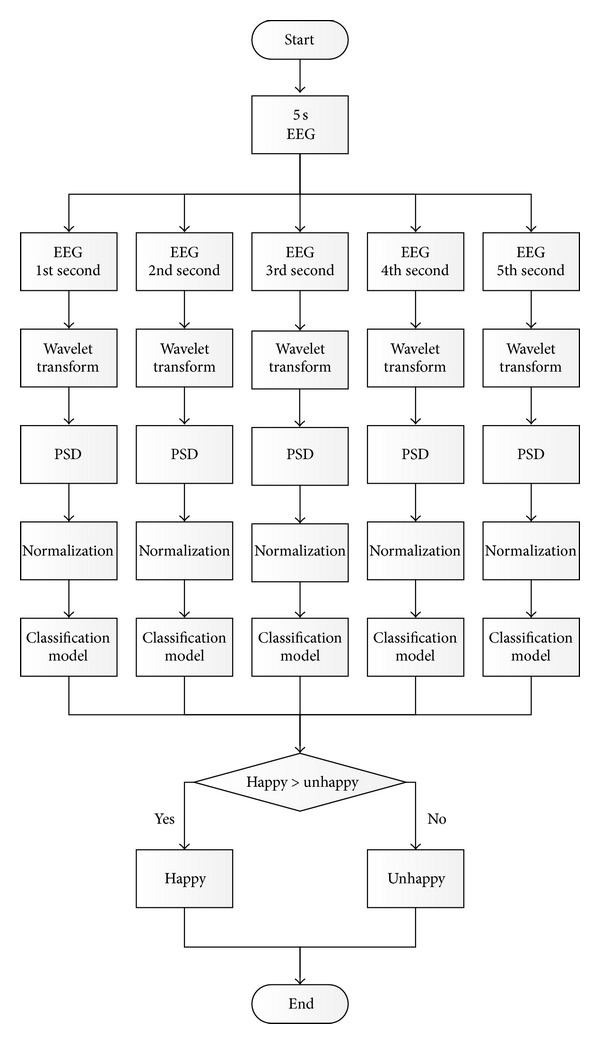
Flowchart of real-time happiness detection system.

**Figure 11:**
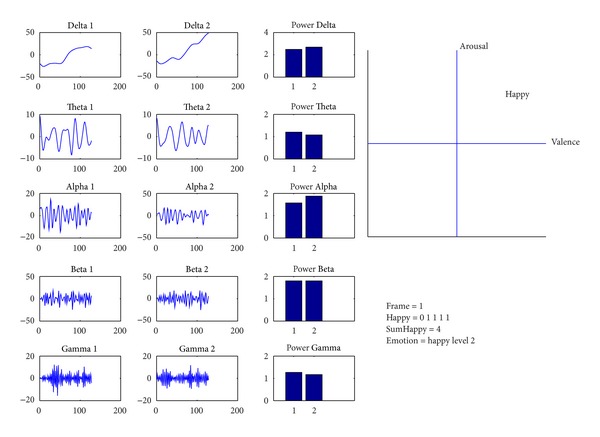
Screenshot of real-time happiness detection system.

From the results of the tests in Section 4, we implement real-time EEG-based happiness detection system using only one pair of channels.  Figure 10 shows the flowchart of the happiness detection system that can be described as follows. The EEG signals with window 1 second are decomposed into 5 frequency bands (i.e., Delta, Theta, Alpha, Beta, and Gamma) by Wavelet Transform. Then we compute PSD of each band as features. With 2 channels, there are 10 features. After that, each feature is normalized by scaling between 0 and 1. Then the normalized features are inserted to classification model, built from previous experiment, to classify emotion. The selected appropriate parameters are derived from LOTO-CV method from previous experiment. The system detects the happy emotion every 5 seconds. Since emotion is classified every second, there are 5 classifications. Majority vote among classifications is used for system detection output. If the number of classifications during consecutive 5 seconds is happy more than unhappy, the detected emotion is happy. Otherwise, the detected emotion is unhappy. We divide the level of emotion from happy to unhappy depending on the number of happy classifications as shown in Table 3. The real-time happiness detection system is implemented using BCI2000 [49] and Matlab as shown in Figure 11. It is run on ASUS K45A with Intel Core i3-3110 M (2.4 GHz, 3 MB L3 Cache).

Furthermore, we develop games for recognizing and controlling happiness that consist of AVATAR and RUNNING. Both games are implemented using UINITY3D based on the real-time happiness detection system that was presented.

**Figure 12:**
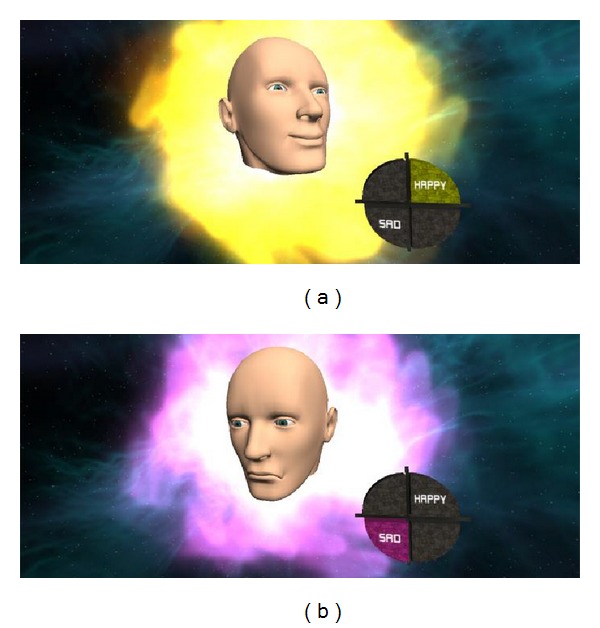
Screenshot of AVATAR game: (a) happy and (b) unhappy.

*AVATAR.* We develop AVATAR game to demonstrate real-time facial expression depending on user's emotion. When the user is happy, the program shows happy face with happy music. Conversely, when the user is unhappy, the program shows unhappy face with unhappy music as shown in Figure 12. This is the game that can help user recognize the happiness.

**Figure 13:**
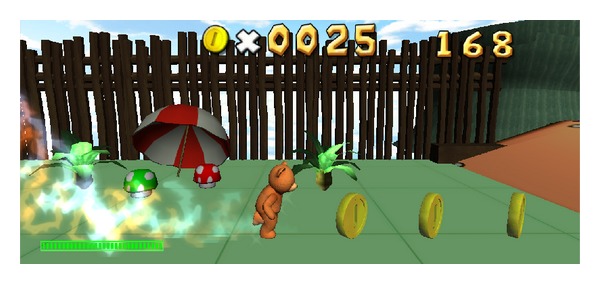
Screenshot of RUNNING game.

*RUNNING*. We develop RUNNING game. The aim of this game is to control the character to run as far as possible within time constraint as shown in Figure 13. The speed of character depends on how happy the user is at the moment. The happier the user is, the more speed the character has. The speed is divided into 6 levels depending on the level of happiness. If the user can sustain their happiness, the character can cover long distance. This is the game that can help user control the happiness.

## 6. Conclusions and Future Work

In this research we propose to use real-time EEG signal to classify happy and unhappy emotions elicited by pictures and classical music. Considering each pair of channels and different frequency bands, temporal pair of channels gives a better result than the other area does, and high frequency bands give a better result than low frequency bands do. All of these are beneficial to the development of emotion classification system using minimal EEG channels in real time. From these results, we implement real-time happiness detection system using only one pair of channels. Furthermore, we develop games to help users recognize and control the happy emotion to be what they want. In the future, we will use other physiological signals such as Galvanic Skin Response (GSR), Electrocardiogram (ECG), and Skin Temperature (ST) combined with EEG to enhance the performance of emotion recognition in the aspect of accuracy and number of emotions. 

## Figures and Tables

**Table 1:** EEG-based emotion recognition researches.

References	Year	Participant	Emotion	Stimulus	Feature	Temporal window	Classifier	Result	Real time
[10]	2006	4 subject-dependent	3 arousal classes	Picture	PSD	—	NB	58%	No
[11]	2008	26 subject-independent	4 classes (joy, anger, sadness, and pleasure)	Music	ASM	1 s	SVM	92.73%	No
[20]	2009	10 subject-dependent	2 valence classes	Picture	CSP	3 s	SVM	93.5%	No
[21]	2009	10 —	3 arousal classes	Recall	PSD	0.5 s	SVM	63%	No
[22]	2009	1 subject-dependent	3 classes (positively excited, negatively excited, and calm)	Picture	statistical features	—	QDA	66.66%	No
[23]	2009	3 subject-dependent	10 classes	Self-elicited	PSD	1 s	KNN	39.97–66.74%	No
[24]	2010	26 subject-independent	4 classes (joy, anger, sadness, and pleasure)	Music	ASM	1 s	SVM	82.29%	No
[25]	2010	6 subject-dependent	2 valence classes 2 arousal classes	Music video	PSD	—	SVM	58.8% (valence) 55.7% (arousal)	No
[26]	2010	26 subject-dependent	4 classes (calm, happy, sad, and fear)	Picture and music	SOM	2 s	KNN	84.5%	No
[28]	2010	15 —	2 classes (calm-neutral and negatively excited)	Picture	HOS	2 s	SVM	82%	No
[29]	2010	12 subject-dependent	2 valence classes 2 arousal classes	Sound	FD	—	threshold	—	Yes
[27]	2011	20 —	5 classes (happy, disgust, surprise, fear, and neutral)	video clip	Entropy	—	KNN	83.04%	No
[31]	2011	6 subject-dependent	2 valence classes	Movie clip	PSD	1 s	SVM	87.53%	No
[32]	2011	20 subject-independent	3 classes (boredom, engagement, and anxiety)	Game	PSD	—	LDA	56%	No
[33]	2011	5 subject-dependent	4 classes (joy, relax, sad, and fear)	Movie	PSD	1 s	SVM	66.51%	No
[34]	2011	11 —	3 valence classes	Picture	ASM	4 s	KNN	82%	No
[30]	2012	27 subject-independent	3 valence classes 3 arousal classes	Video	PSD and ASM	—	SVM	57.0% (valence) 52.4% (arousal)	No
[35]	2012	32 —	2 valence classes 2 arousal classes	Music video	PSD and ASM	—	NB	57.6% (valence) 62.0% (arousal)	No
[36]	2012	20 subject-dependent	5 classes (happy, angry, sad, relaxed, and neutral)	Picture	FD	—	SVM	70.5%	Yes
[37]	2012	5 subject-dependent	3 classes (positively excited, negatively excited, and calm)	Picture	HOC	—	KNN	90.77%	No
[38]	2012	4 —	2 valence classes 2 arousal classes	Video clip	ASP	—	—	66.05% (valence) 82.46% (arousal)	No
[39]	2012	32 —	2 classes (stress and calm)	Music video	PSD	—	KNN	70.1%	No
[40]	2012	36 —	3 classes	Music video	PSD	—	ANN	—	Yes
[41]	2013	11 subject-independent	2 valence classes	Picture	PSD	4 s	SVM	85.41%	No

*The feature, temporal window, and classifier shown in this table are the sets giving the best accuracy of each research.

Feature: Power Spectral Density (PSD), Spectral Power Asymmetry (ASM), Common Spatial Pattern (CSP), Higher Order Crossings (HOC), Self-Organizing Map (SOM), Higher Order Spectra (HOS), Fractal Dimension (FD), and Asymmetric Spatial Pattern (ASP).

Classifier: Support Vector Machine (SVM), Naïve Bayes (NB), Quadratic Discriminant Analysis (QDA), K-Nearest Neighbors (KNN), Linear Discriminant Analysis (LDA), Multilayer Perceptron (MLP), and Artificial Neural Network (ANN).

**Table 2:** EEG signal decomposition.

Frequency band	Frequency range (Hz)	Frequency bandwidth (Hz)	Decomposition level
Delta	0–4	4	A4
Theta	4–8	4	D4
Alpha	8–16	8	D3
Beta	16–32	16	D2
Gamma	32–64	32	D1

**Table 3:** Level of happiness.

Happy	Unhappy	Emotion
0	5	Unhappy level 3
1	4	Unhappy level 2
2	3	Unhappy level 1
3	2	Happy level 1
4	1	Happy level 2
5	0	Happy level 3
